# Expression Patterns of Glutathione Transferase Gene (*GstI*) in Maize Seedlings Under Juglone-Induced Oxidative Stress

**DOI:** 10.3390/ijms12117982

**Published:** 2011-11-16

**Authors:** Hubert Sytykiewicz

**Affiliations:** Department of Biochemistry and Molecular Biology, University of Natural Sciences and Humanities, B. Prusa 12 Street, 08-110 Siedlce, Poland; E-Mail: huberts@uph.edu.pl; Tel.: +48-25-643-1298; Fax: +48-25-643-1367

**Keywords:** juglone, glutathione transferase gene, real-time quantitative RT-PCR, gene expression, oxidative stress, phytotoxicity, maize

## Abstract

Juglone (5-hydroxy-1,4-naphthoquinone) has been identified in organs of many plant species within *Juglandaceae* family. This secondary metabolite is considered as a highly bioactive substance that functions as direct oxidant stimulating the production of reactive oxygen species (ROS) in acceptor plants. Glutathione transferases (GSTs, E.C.2.5.1.18) represent an important group of cytoprotective enzymes participating in detoxification of xenobiotics and limiting oxidative damages of cellular macromolecules. The purpose of this study was to investigate the impact of tested allelochemical on growth and development of maize (*Zea mays* L.) seedlings. Furthermore, the effect of juglone-induced oxidative stress on glutathione transferase (*GstI*) gene expression patterns in maize seedlings was recorded. It was revealed that 4-day juglone treatment significantly stimulated the transcriptional activity of *GstI* in maize seedlings compared to control plants. By contrast, at the 6th and 8th day of experiments the expression gene responses were slightly lower as compared with non-stressed seedlings. Additionally, the specific gene expression profiles, as well as the inhibition of primary roots and coleoptile elongation were proportional to juglone concentrations. In conclusion, the results provide strong molecular evidence that allelopathic influence of juglone on growth and development of maize seedlings may be relevant with an induction of oxidative stress in acceptor plants.

## 1. Introduction

Juglone (5-hydroxy-1,4-naphthoquinone) is an allelochemical that has been isolated from tissues and organs of many plant species belonging to *Juglandaceae* family [1–3]. In living plants, juglone naturally occurs mainly as a non-toxic glycosylated form (hydrojuglone β-d-glucopyranoside), but when exposed to the air or soil components, this allelochempound is immediately transformed into the oxidized and highly toxic form [4,5]. Allelopathic potential of this walnut constituent leads to inhibition of seed germination and growth of susceptible acceptor plants [6–10]. Detrimental impact of this naphthoquinone may be associated with suppressing the intensity of a wide range of physiological processes and biochemical reactions occurring in plant tissues [9,11,12]. It has been reported that juglone is responsible for reducing chlorophyll content [13], disrupting root plasma membrane and decreasing of water uptake [14], inhibition of photosynthesis, transpiration, respiration and stomatal conductance [15,16]. One of the most important mechanisms underlying the phytotoxic influence of juglone is involved in the pro-oxidation action within tissues of targeted plants [17,18]. It is noteworthy, that the cytotoxic effects of this allelochemical are not only restricted towards plants but also include a large spectrum of antiviral, antibacterial, antifungal and nematicidal activities [3,19–21]. Furthermore, the anti-carcinogenic action of this allelochemical has been recently reported [22–24].

Plants have evolved a sophisticated and highly complex network of interacting regulatory mechanisms that participate in limiting of cellular oxidative damages in stressed organs. This multi-level antioxidant machinery embraces both antioxidant enzymes and metabolites responsible for reactive oxygen species (ROS) scavenging. Cytosolic glutathione transferases (GSTs, E.C.2.5.1.18) play the crucial role in maintaining the redox homeostasis [25–27]. The most essential biological functions of GSTs are associated with mediating the conjugation of electrophillic xenobiotics with glutathione (γ-Glu-Cys-Gly; GSH) and providing protection against oxidative burst spreading throughout plant tissues [28–30]. Furthermore, plant GSTs are involved in signal transduction, non-catalytic binding of flavonoids, and participate in intermediary metabolism [31]. It should be underlined that overexpressing the specific isoenzymes of GSTs in transgenic plants resulted in enhanced tolerance to herbicide and oxidative stress [31–33].

There are limited biochemical studies concerning ROS production within plant tissues subjected to allelopathic stressors [34–37]. However, the molecular level of this interconnection still remains unclear. It is hypothesized that specific reconfigurations in *GstI* gene expression patterns reflect the levels of oxidative burst in plant tissues under juglone-triggered allelochemical stress. Gene expression profiling using the real-time qRT-PCR technique has become a precise and powerful molecular tool in ecotoxicological experiments nowadays. Therefore, the ultimate purpose of performed studies was to gain insight into the molecular responses of antioxidant *GstI* gene within stressed maize seedlings, as the model plant. Verifying the hypothesis has been carried out in two subsequent phases: (1) assessment the influence of juglone-induced oxidative stress on seed germination, post-germinative growth and development parameters of maize seedlings; (2) comparative analysis of *GstI* gene expression specific profiles between experimental groups of juglone-treated maize seedlings and the control. Furthermore, it was investigated whether the nature of these alternations might be associated with a dose- and time-dependent juglone exposure.

## 2. Results and Discussion

### 2.1. The Impact of Juglone Treatments on Seed Germination and Post-Germinative Growth of Maize

**Figure 1:**
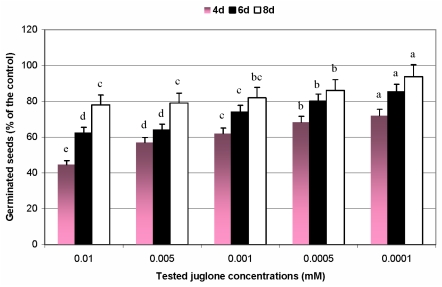
Effects of different juglone treatments on germination process of maize seeds. Values denoted with different letters are statistically significant at *p* < 0.01 (Duncan’s multiple range test); 100%—number of germinated maize seeds in the control group; d—day of the experiment.

It has been found that germination process of maize seeds was significantly suppressed under juglone-induced oxidative stress (Figure 1). The strongest inhibitory effect of the tested allelochemical was observed at the 4th day of biotests, and in the next four days of conducted experiments the capacity of seeds to germination slightly and gradually increased. Furthermore, it was estimated that increase in juglone concentrations evoked the proportional decline in number of germinated seeds when compared to the non-stressed control. The highest inhibition of assessed parameter was noted at 0.01 mM concentration of juglone (4d: 55%, 6d: 38%, 8d: 22%). On the contrary, the lowest concentration of the allelochemical resulted in less suppression of seed germination (4d: 28%, 6d: 15%, 8d: 6%).

**Figure 2:**
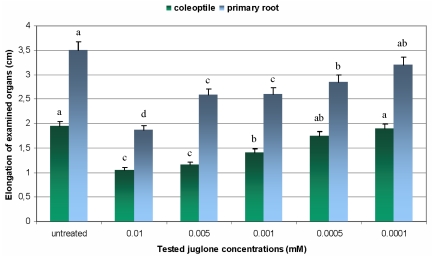
Elongation of coleoptiles and primary roots of juglone-stressed maize seedlings. Values denoted with different letters are statistically significant at *p* < 0.01 (Duncan’s multiple range test). Parameters were measured at the 8th day of the experiments.

The results regarding the influence of different juglone treatments on elongation of coleoptiles and primary roots of maize seedlings are displayed in Figure 2. It was revealed that all tested amounts of the allelochemical possessed deleterious effect on growth of examined maize organs in relation to untreated plants. Additionally, it was observed that the degree of growth inhibition was dependent on juglone dose and treatment duration. Maximal decrease in elongation of coleoptiles and primary roots of maize seedlings was observed at 0.01 mM juglone treatment, whereas the lowest suppression of the growth was recorded at 0.0001 mM concentration of the tested phytotoxin. The highest amount of juglone (0.01 mM) evoked the similar inhibitory effect on length of maize organs (46 and 47% inhibition of coleoptiles and primary roots elongation, respectively). It should be underlined that lower treatment concentrations differentially interfered with the growth parameters of examined organs. Coleoptiles elongation was significantly more suppressed by 0.005 and 0.001 mM juglone in relation to primary roots growth. In contrast, primary roots elongation was more intensively inhibited at 0.0005 and 0.0001 mM amounts of this allelochemical when compared with recorded parameter of coleoptiles.

**Figure 3:**
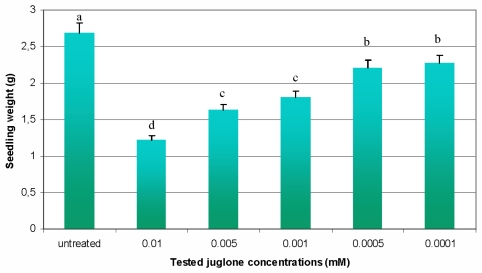
Influence of juglone on maize seedling weight. Values denoted with different letters are statistically significant at *p* < 0.01 (Duncan’s multiple range test). Parameter was recorded at the 8th day of the experiments.

The performed biotests indicated that all tested juglone treatments evoked a decline in maize seedling weight (Figure 3). The obtained results revealed that the maximal inhibition (55%) of measured parameter occurred at the highest concentration of juglone (0.01 mM), while the minimal decrease (16%) in seedling weight was observed at 0.0001 mM of the analysed allelochemical. Furthermore, statistical analyses proved the significance of differences (*p* < 0.01) between the mean values of seedling weight in experimental groups and the control plants of maize.

Statistical analyses confirmed that there is a strong negative correlation (*p* < 0.01) between estimated parameters of maize growth and development (seed germination, elongation of coleoptiles and primary roots, seedling weight) and tested juglone concentrations (Table 1).

Until now, it has been published a limited number of analyses concerning the impact of juglone on growth and development of monocotyledonous acceptor plants [9,10,14,38,39]. Hence, the present work adds a piece of evidence, based on *in vitro* experiments that juglone possessed the strong inhibitory influence on seed germination and elongation of coleoptiles and primary roots of maize seedlings. It has been recently reported that 0.01 mM juglone treatment suppressed the number of germinated seeds of *Triticum aestivum* (L.) in relation to the untreated control [39]. Interestingly, it was also observed that germination process of the winter wheat seeds was completely arrested at 1.0 mM juglone concentration. Furthermore, it should be underlined that Matok [39] recorded a decline (approx. 30%) in shoot elongation and overall inhibition of primary root development of winter wheat seedlings treated with 0.01 mM juglone. According to Hejl and Koster [14], the growth of maize seedlings was significantly repressed by juglone exposure (13% at 0.01 mM and 21% at 0.1 mM) when compared to non-stressed plants. The performed biotests demonstrate significant reduction in maize seedlings weight under juglone-induced stress. Similar tendency was observed by Jose and Gillespie [15]. These authors documented the phytotoxic impact of juglone on weight of 6-day maize seedlings growing under hydroponic culture. It was evidenced that 72-h juglone treatment at 0.01 mM concentration inhibited shoot and root relative growth rates (32% and 65% reduction, respectively). Additionally, Hejl and Koster [14] have also revealed a significant decrease in biomass of juglone-stressed maize seedlings in relation to the non-treated control. Matok [39] has also recorded that 0.01 mM juglone treatment resulted in suppression of above-ground parts of winter wheat seedlings weight in comparison with untreated plants.

### 2.2. Expression of GstI Gene in Juglone-Stressed Maize Seedlings

**Figure 4:**
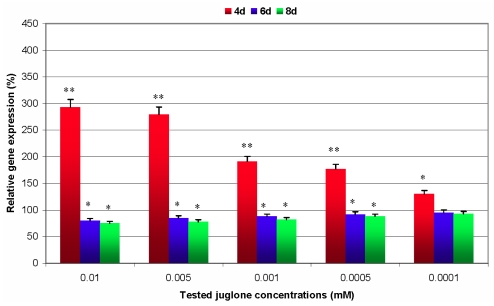
Juglone-induced changes in levels of relative expression of *GstI* gene in coleoptiles of maize seedlings. 100%—the reference level of gene expression in untreated maize seedlings; * *p* < 0.05, ** *p* < 0.01 (Student’s *t*-test)—significant differences in relative gene expression between the tested treatment group and the control group of seedlings.

**Figure 5:**
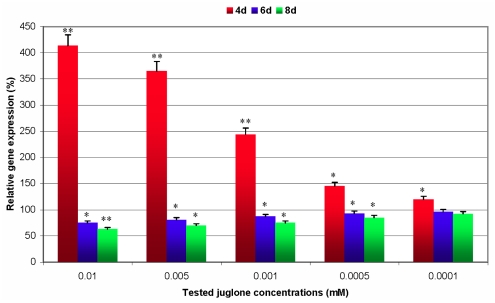
Transcript levels of *GstI* gene in primary roots of maize seedlings affected by juglone treatment. 100%—the reference level of gene expression in untreated maize seedlings; * *p* < 0.05; ** *p* < 0.01 (Student’s *t*-test) —significant differences in relative gene expression between the tested treatment group and the control group of seedlings.

Quantitative data of *GstI* gene expression in coleoptiles and primary roots of juglone-treated maize seedlings are presented in Figures 4 and 5. Transcript levels of analysed gene in tested groups of plants were calculated as percentage changes in relation to the control organs. It was demonstrated that 4-day juglone treatment significantly stimulated the relative expression of *GstI* in all examined groups of maize seedlings when compared to non-stressed plants. It should be noted that changes in levels of the gene expression were proportional to allelochemical concentrations. The highest increase in the transcript accumulation was affected by 0.01 mM amount of juglone (2.9-fold enhancement in coleoptiles and 4.2-fold in primary roots, respectively). On the other hand, the lowest induction of analysed gene expression was noted at 0.0001 mM juglone concentration (1.3-fold increase in coleoptiles and 1.2-fold in primary roots, respectively). Transcriptional responses of *GstI* gene in primary roots were more distinct in case of three treatments of juglone (0.01, 0.005 and 0.0001 mM) when compared to the treated samples of coleoptiles. Conversely, 6- and 8-day juglone treatments affected a significant decrease in *GstI* gene expression in all groups of maize seedlings in relation to the control. The degree of this decline was the most explicit in primary roots of maize seedlings comparing to coleoptiles. The most considerable decline in the transcript levels was estimated at the highest concentration of analysed allelochemical (20% decrease in *GstI* gene expression in coleoptiles at the 6 days and 25% at the 8th day; 25% diminution in expression levels in primary roots at the 6th day and 37% decrease at the 8th day). Performed statistical analyses proved the significance (*p* < 0.05) of decline in expression of analysed gene, with the exception of 0.0001 mM juglone treatment after 6 and 8 days of experiments. Additionally, there were no significant differences in *GstI* transcript levels in examined organs of non-stressed maize seedlings between the 4th and 8th day of biotests.

This study was designed to investigate the *in vitro* molecular responses of antioxidant *GstI* gene under juglone-induced oxidative stress in plant tissues. Maize seedlings were used as model to test the hypothesis whether there are specific patterns of alternations in analysed gene expression affected by different allelochemical treatments and exposure duration. Glutathione transferases embrace a complex array of enzymes involved with the metabolic response of plant tissues to many environmental stressors [28,40,41]. Catalytic functions of GSTs enzymes are connected with the phase II-mediated detoxification of a wide spectrum of xenobiotics and endogenous toxic substances. Secondarily, cytoprotective properties of GSTs are also associated with limiting the level of oxidative stress by scavenging of ROS in plants [29,42,43].

The isoenzyme GST I occurring in maize tissues belongs to the type I [44] and class *phi* [45] within the large and diverse family of GSTs. The biochemical properties and biological functions of this isoenzyme isolated from maize has been intensively studied [44]. According to Karavangeli *et al.* [42], the activity of maize GST I isoenzyme in transgenic tobacco plants was significantly stimulated under chloroacetanilide herbicide alachlor treatment when compared to the control. Jiang and Yang [46] investigated the oxidative-inducing effect of prometryne (s-triazine selective herbicide) in wheat seedlings. These authors revealed a dose-dependent significant increase in the activity of *GST* and up-regulation of *GST* gene expression in prometryne-treated plants in relation to the non-stressed control. The similar results were obtained by Pašková *et al.* [47]. These authors indicated that tested homocyclic aromatic hydrocarbons (phenanthrene, anthracene, fluorine) and their *N*-heterocyclic derivates considerably enhanced the activity of GST in *T. aestivum* plants. According to McGonigle *et al.* [44], the application of DNA microarray analysis revealed that dichlormid treatment resulted in overexpression of 15 transcripts level of *GST* genes in etiolated maize seedlings when comparing to the control. Furthermore, it was ascertained that 18 *GST* genes were up-regulated in the presence of ethanol. It should be emphasized that Jain *et al.* [48] have identified 23 transcripts of *GST* genes that were accumulated and 4 genes that were down-regulated in Bala (arsenate-tolerant) rice seedlings subjected to 7-day arsenate treatment.

In this report, the allelopathic influence of tested allelochemical on seed germination and post-germinative processes of growth and development of maize plants was confirmed. The observed suppression of recorded growth parameters occurred in parallel with characteristic reconfigurations in *GstI* gene expression in examined organs of maize seedlings (coleoptiles and primary roots), subjected to different juglone treatments. It has been revealed that 4-day juglone treatment leads to a significant overexpression of analysed gene, whereas at the 6th and 8th day of the experiments a slight down-regulation of *GstI* was recorded. Additionally, this study demonstrates a critical role a dose- and time-dependent juglone exposure on transcriptional activity of analysed gene within stressed plants in comparison with the control seedlings. According to Akbulut and Cakir [28], numerous abiotic and biotic stimuli possess the ability to initiate the generation of ROS in plants. Excessive oxidative burst in stressed tissues may lead to many cytotoxic effects regarding the oxidation of bioactive macromolecules (DNA, proteins and lipids). Effective adaptation of plants to stressful exogenous factors depends on the relevant coordination of various elements of highly complex antioxidative system that restraint the detrimental level of oxidative damages [49]. Mylona *et al.* [27] claim that successful protection of plant cells under xenobiotic treatment is based on both detoxification and antioxidant responses. It is highly probable, that demonstrated overexpression of *GstI* gene in both investigated organs of maize seedlings at the 4th day of experiments enhances the tolerance of stressed plants towards the influencing allelochemical. Interestingly, according to Jiang and Yang [46], the functioning of antioxidative defense mechanisms may be considerably disturbed under excessive ROS production. Therefore, it is likely that the recorded decline in *GstI* gene expression occurring after 6 and 8 days of permanent juglone exposure is a circumstantial evidence of breakdown in the antioxidant defense systems in stressed maize seedlings. The obtained results highlight the molecular basis of maize *GstI* gene expression reconfigurations during juglone-induced oxidative stress. However, further studies are required to gain more detailed insight into molecular responses of antioxidant genes under the long-term allelochemical stress in acceptor plants.

## 3. Experimental Section

### 3.1. Reagents

Juglone (5-hydroxy-1,4-naphthoquinone, m.w. 174.15) was purchased from Sigma Aldrich (Poznań, Poland). All chemicals reagents used in this study were molecular biology grade.

### 3.2. Plant Material and Treatment Conditions

**Figure 6:**
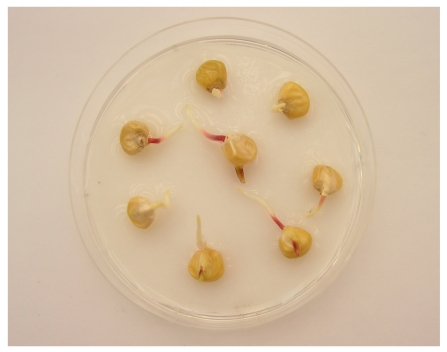
An illustrative example for maize growth and development experiments under juglone-stressed conditions.

*Zea mays* (L.) cv. Złota Karłowa was used in these studies. Maize seeds were surface sterilized in 70% ethanol for 2 min, 0.1% mercuric chloride for 3 min, and then thoroughly rinsed with deionized water (5 times for 30 s). After this procedure, samples of eight seeds were placed on filter paper (Whatman No. 1) in sterile plastic Petri dishes (9 cm diameter), as shown in Figure 6. The experiments were completely randomized with three independent replications (*N* = 200 seeds during the single experiment) per each juglone treatment. Tested concentrations of juglone (0.01, 0.005, 0.001, 0.0005 and 0.0001 mM) were prepared by dissolving this allelochemical in methanol-deionized water solution (4.0% v/v). The filter paper discs were moistened with 2 mL of a 4% methanolic aqueous solution of tested concentrations of juglone, whereas controls were treated with 2 mL of methanol-deionized water solution (4.0% v/v). Petri dishes containing tested seeds were incubated in a growth chamber at 22 °C ± 2 °C/16 ± 2 °C (day/night), relative humidity of 65 ± 5%, light intensity of 100 μM·m^−2^·s^−1^, and a 16-h-light/8-h-dark cycle. Before each experiment, the growth chamber was sterilized with the use of UVC lamp for 12 h. Four-, six- and eight-day-old maize seedlings were harvested, and subsequently the length of primary roots and coleoptiles [cm] and seedling weight [g] were measured. Additionally, at termination of each biotest, the number of germinated seeds was recorded.

### 3.3. Total RNA Isolation and Quantification

Total RNA was isolated fom 10 randomly selected juglone-treated and non-stressed (control) maize seedlings. For each sample preparation, 100 mg of collected coleoptiles and primary roots were ground immediately with liquid nitrogen. Total RNA was extracted using Spectrum^TM^ Plant Total RNA Kit (Sigma Aldrich, Poznań, Poland) and isolated RNA was cleaned with On-Column DNase I Digestion Set (Sigma Aldrich, Poznań, Poland). The quantification of RNA samples was determined using a *NanoVue* spectrophotometer (GE Healthcare). Furthermore, A_260/280_ and A_260/230_ ratios were calculated in order to establish the sample integrity and assess the degree of contamination of proteins or other organic components. The intact RNA samples possessing the high integrity were accepted for further molecular analyses.

### 3.4. cDNA Synthesis

After DNA digestion, 1 μg of total RNA samples was used for a reverse-transcription reaction with the application of RevertAid^TM^ Premium First Strand cDNA Synthesis Kit (Fermentas) following the manufacturer’s instructions. Synthesis of the first strand cDNA was performed using oligo (dT)_18_ primers. The following negative control reactions were used to verify the results of the first strand cDNA synthesis: RT-negative (all reagents for the reverse transcription reaction except for the RevertAid^TM^ Premium Enzyme Mix) and NTC (no template control).

### 3.5. Gene Expression Analysis

Specific expression patterns of maize *GstI* gene (accession number: M16902.1, GenBank) were analysed using real-time qRT-PCR (real-time quantitative Reverse Transcription-PCR). Glyceraldehyde-3-phosphate dehydrogenase (GAPDH) was used as a housekeeping reference gene (accession number: X07156.1, GenBank). Real-time detection of fluorescence was performed on the LightCycler 480 II (Roche) equipped with the specialist software (LCS480 1.5.0.39.). All reactions were carried out in a 96-well microplates using 10 μL of FastStart Universal Probe Master (Roche), 1 μL of forward primer mix (500 nM), 1 μL of reverse primer mix (500 nM), 0.6 μL of TaqMan^®^ fluorescent probe (300 nM), 5 μL of template cDNA and 2.4 μL of sterile and RNase-free water. Primers and probes were purchased in TIB-MOLBIOL (Poland, Poznań) and their sequences are listed in Tables 2 and 3. The thermal cycling parameters were set at 95 °C for 10 min for 1 cycle (activation of *FastStart Taq* DNA Polymerase), and 40 cycles at 95 °C for 20 s (denaturation) and 60 °C for 60 s (amplification and *real-time* analysis). After completion of the reactions, the cycle threshold (C_T_) value was calculated and subsequently, the ΔΔC_T_ algorithm was used [50]. The final value of relative gene quantification was expressed as *n*-fold change in *GstI* transcript level in tested samples in comparison with the controls. The results are presented as the mean ± standard deviation (SD) of three independent analyses.

### 3.6. Statistical Analysis

All data were expressed as the mean ± standard deviation (SD) of three independent replicates (*n* = 3). The obtained results were analysed by using STATISTICA 9.0 software (StatSoft Poland). Significance of differences between mean values of tested parameters (seed germination, seedling weight, primary root and coleoptile elongation) of juglone-treated maize seedlings and control plants were subjected to one-way analysis of variance (ANOVA). Interdependence between tested juglone concentrations and recorded parameters of maize growth was analysed by calculation of the Pearson’s correlation coefficient. Student’s *t*-test was used to assess the significance of differences in *GstI* gene expression levels in primary roots and coleoptiles between tested groups of seedlings and the control plants (non-stressed with juglone). Values of *p* < 0.05 were considered statistically significant.

## 4. Conclusions

In summary, the presented results provide strong molecular evidence that allelopathic effects of juglone on growth and development of the maize seedlings may be associated with triggering the detrimental level of oxidative burst in stressed plant tissues. Furthermore, the work reveals that the scale and character of the molecular responses of antioxidant *GstI* gene in stressed acceptor plants reflect the exposure duration and dose of juglone treatment. However, more detailed studies are needed to uncover the mechanisms of transcriptional gene regulation involved with molecular-level changes occurring in juglone-treated plants.

## Figures and Tables

**Table 1:** Values of Pearson’s correlation coefficient calculated between tested juglone concentrations and recorded parameters of maize growth and development. ** *p* < 0.01.

Seed germination (% of the control)	Elongation of primary roots	Elongation of coleoptiles	Seedling weight
−0.925 **	−0.960 **	−0.905 **	−0.965 **

**Table 2:** Sequences of primers designed for amplification of *GstI* and *GAPDH* genes. F—forward primer, R—reverse primer, *T*_m_—melting temperature.

Amplified gene	Type of Primer	Primer Sequence
*GstI*	F	CGGTGACTTGTACCTCTTCGAATC
	R	ATCCACCATTGCTGCCTCC
*GAPDH*	F	AAGCCGGTCACCGTCTTT
	R	CATCTTTGCTTGGGGCAGA

**Table 3:** Sequences of *TaqMan* ^®^ fluorescent probes designed for amplification of *GstI* and *GAPDH* genes. 6-FAM—6-carboxyfluorescein, BBQ—BlackBerry Quencher.

Amplified gene	*TaqMan*^®^ Probe Sequence
*GstI*	6FAM-TCCCTCAACAGCTCTGGCTTGTTTTT-BBQ
*GAPDH*	6FAM-CTTCACTGACAAGGACAAGGCTGCT-BBQ
